# Intervention effect estimates in randomised controlled trials conducted in primary care versus secondary or tertiary care settings: a meta-epidemiological study

**DOI:** 10.1186/s12874-022-01815-2

**Published:** 2022-12-22

**Authors:** Amandine Dugard, Elsa Tavernier, Agnès Caille, Agnès Dechartres, Adeline Hoang, Bruno Giraudeau, Clarisse Dibao-Dina

**Affiliations:** 1 University of Tours, University of Nantes, INSERM 1246-SPHERE, Tours, France; 2 INSERM CIC1415, CHRU de Tours, Tours, France; 3 Sorbonne Université, INSERM, Institut Pierre Louis d’Epidémiologie et de Santé PubliqueAP-HP. Sorbonne Université, Hôpital Pitié Salpêtrière, Département de Santé Publique, F75013 Paris, France; 4 Department of General Practice, University of Tours, Tours, France

**Keywords:** Meta-epidemiological study, Cochrane systematic review, Primary care

## Abstract

**Background:**

Many clinical practice guidelines are based on randomised controlled trials conducted in secondary or tertiary care setting and general practitioners frequently question their relevance for primary care patients. Our aim was to compare the intervention effect estimates between primary care setting randomised controlled trials (PC-RCTs) and secondary or tertiary care setting randomised controlled trials (ST-RCTs).

**Methods:**

Meta-epidemiological study of meta-analyses (MAs) of a binary outcome including at least one PC-RCT and one ST-RCT. PC-RCTs were defined as trials recruiting patients in general practices, primary care practices, family practices, community centers or community pharmacies. ST-RCTs were defined as trials recruiting in hospitals, including hospitalized patients, hospital outpatients and patients from emergency departments. For each MA, we estimated a ratio of odds ratio (ROR) by using random-effects meta-regression, with an ROR less than 1 indicating lower estimates of the intervention effect in PC-RCTs than ST-RCTs. Finally, we estimated a combined ROR across MAs by using a random-effects meta-analysis. We performed subgroup analyses considering the type of outcomes (objective vs subjective), type of experimental intervention (pharmacological vs non-pharmacological), and control group (active vs inactive) as well as analyses adjusted on items of the Cochrane Risk of Bias tool.

**Results:**

Among 1765 screened reviews, 76 MAs with 230 PC-RCTs and 384 ST-RCTs were selected. The main medical fields were pneumology (13.2%) and psychiatry or addictology (38.2%). Intervention effect estimates did not significantly differ between PC-RCTs and ST-RCTs (ROR = 0.97, 95% confidence interval 0.88 to 1.08), with moderate heterogeneity across MAs (I^2^ = 45%). Subgroup and adjusted analyses led to consistent results.

**Conclusion:**

We did not observe any significant difference in intervention effect estimates between PC-RCTs and ST-RCTs. Nevertheless, most of the medical fields in this meta-epidemiological study were not representative of the pathologies encountered in primary care. Further studies with pathologies more frequently encountered in primary care are needed.

**Supplementary Information:**

The online version contains supplementary material available at 10.1186/s12874-022-01815-2.

## Background

Many studies have highlighted the lack of adherence to clinical practice guidelines by general practitioners (GPs) [1, 2]. GPs generally argue a lack of relevance or difficulties in applying guidelines [3, 4], and they particularly express concerns about the applicability of clinical trial results used to establish guidelines to their own patients [5, 6]. The first reason is that many randomised controlled trials (RCTs) include highly selected patients, which restricts the generalizability of their results [7]. As an example, a study found that less than half of primary-care patients would have been included in hypertension trials [8]. The second, highly related to the first one reason is that in most RCTs, patients are recruited in secondary- or tertiary-care settings [9] rather than in primary-care. However, as compared with secondary- or tertiary-care setting patients, primary-care patients have less severe disease and more undifferentiated symptoms [10] but more multi-morbidity [11]. Such differences may lead to differences in benefits and harms of the intervention. Our hypothesis is that intervention effect estimates differ between primary care setting randomised controlled trials (PC-RCTs) and secondary or tertiary care setting RCTs (ST-RCTs).

We performed a meta-epidemiological study to assess whether intervention effect estimates differ between PC-RCTs and ST-RCTs.

## Methods

This was a meta-epidemiological study, a study design used to compare intervention effect estimates between trials with and without a characteristic of interest [12]. In this study, we focused on the setting, with the aim of comparing PC-RCTs to ST-RCTs. A meta-epidemiological study is performed by generally using a two-step approach and, contrary to traditional epidemiological studies, units of analysis are studies rather than patients [13]. First, for each selected MA, we assessed the difference in intervention effects between studies with the characteristics of interest (i.e., primary care setting) and without the characteristics of interest. This step involved using a meta-regression for each selected MA (with the intervention effect considered the outcome and the characteristic of interest the independent variable). Second, results from the meta-regression were meta-analysed over the different MAs.

### Search strategy

On July 9, 2020, we searched the Cochrane Database of Systematic Reviews to retrieve all systematic reviews with meta-analyses (MAs) that were published, with no restriction on time, by using the following text words in the full text: “primary care” OR “primary healthcare” OR “general practice” OR “family practice”. We only used primary care keywords to ensure that we had PC-RCTs in each MA.

### Selection of relevant MAs and trials

We screened the full text of potentially eligible systematic reviews to select MAs of binary outcomes that had at least 3 trials (3 studies is the minimum to perform a meta-regression) and at least one PC-RCT and one ST-RCT. PC-RCTs were defined as trials recruiting patients in general practices, primary care practices, family practices, community centers or community pharmacies according to the definition by Afonso et al. [14]. Trials in nursing homes or at home were not considered PC-RCTs and were excluded. ST-RCTs were defined as trials recruiting in hospitals, including hospitalized patients, hospital outpatients and patients from emergency departments. Hospital-at-home trials were not considered ST-RCTs and were excluded. Trials with both primary and secondary or tertiary care settings were excluded, as were trials with an unclear setting or trials including patients in other than primary or secondary or tertiary care settings, such as schools. For this study, we selected only RCTs and excluded non-randomised or quasi-randomised trials.

If more than one MA was eligible within the same review, we selected the MA for the primary efficacy outcome, then the MA with the highest number of trials. MAs of adverse events were not included because of the uncertainty in the direction of bias. We also discarded MAs when it was impossible to determine which group was the experimental and control group*.*

When a trial was included several times within the same MA, we kept only the duplicate with the largest sample size. When a trial was included in several selected MAs, we kept the one in the most recent systematic review.

All this selection process was performed independently by two reviewers (A.D., A.H.), with disagreements resolved by discussion, referring to a third opinion (C.D.D.) when necessary.

### Data collection

Two independent reviewers (A.D., A.H.) collected data from all selected MAs and trials by using a standardised data collection form. Disagreements were resolved by discussion, referring to a third opinion (C.D.D.) when necessary. The following characteristics were extracted:

*For each meta-analysis:*

- General information: year of publication, first author name
- Medical field
- Type of intervention (pharmacological, non-pharmacological), type of comparator (active control or inactive control, with inactive control defined as placebo or no added intervention to usual care, sham or other)
- Outcome and whether it was subjective or objective following the classification provided by Savović et al. [15]
  - All-cause mortality
  - Objectively assessed (e.g., laboratory results)
  - Objectively assessed but potentially influenced by a clinician or patient (e.g., smoking cessation)
  - Subjectively assessed (e.g., pain)
    - Objective outcomes were the two first categories and subjective outcomes were the two last categories.

- Number of included studies

*For each eligible RCT included in the MA:*

- Author
- Year of publication
- First author name
- Trial type (PC-RCT or ST-RCT)
- Number of centers (for primary care, a center was defined as a practice, and for secondary and tertiary care, it was a hospital)
- Sample size
- For each group, the number of events and the number of patients analysed
- Domains of the Cochrane Risk of Bias tool [16] as was reported by the authors of the systematic reviews

*For PC- RCTs, we extracted the following additional information if mentioned in the MA:*

- Type of primary care setting
- Healthcare professionals who recruited patients (when mentioned)
- Whether the settings where patients are seen during the study for recruitment, follow-up and primary outcome assessment were the same or not
- Country(ies) where the study was performed because of existing differences in healthcare systems between countries in primary-care

All data were collected from the systematic review report except for the number of events, which was collected from the trial reports when missing in the MA report.

### Statistical analysis

We estimated intervention effects as odds ratios (OR). Outcome events were re-coded so that an OR less than 1 indicated a beneficial effect of the experimental intervention. Randomised controlled trials with no event in both groups did not contribute to the analysis.

### Meta-epidemiological analysis

To compare PC-RCTs and ST-RCTs, we used the two-step approach described by Sterne et al. [12]. First for each meta-analysis, we estimated a ratio of odds ratio (ROR) by using random-effects meta-regression. In our study, the ROR was the ratio of the OR for PC-RCTs to the OR for ST-RCTs, An ROR less than 1 indicates lower intervention effects for PC-RCTs. Second, we estimated a combined ROR across meta-analyses and the 95% confidence interval (CI) by using a random-effects meta-analysis model. The heterogeneity across MAs was assessed with the I^2^ statistic and its 95% CI and the between–meta-analysis variance τ^2^.

### Subgroup and sensitivity analyses

Subgroup analyses were planned according to the objectivity of outcomes, type of intervention in the experimental group (pharmacological vs non-pharmacological), and control group (active vs inactive). We used an interaction test to assess whether the combined ROR varied between subgroups. We also performed sensitivity analyses by adjusting meta-regression models on each item of the Risk of Bias tool [16] (high or unclear risk vs low risk).

## Results

### Study selection and general characteristics

**Fig. 1:**
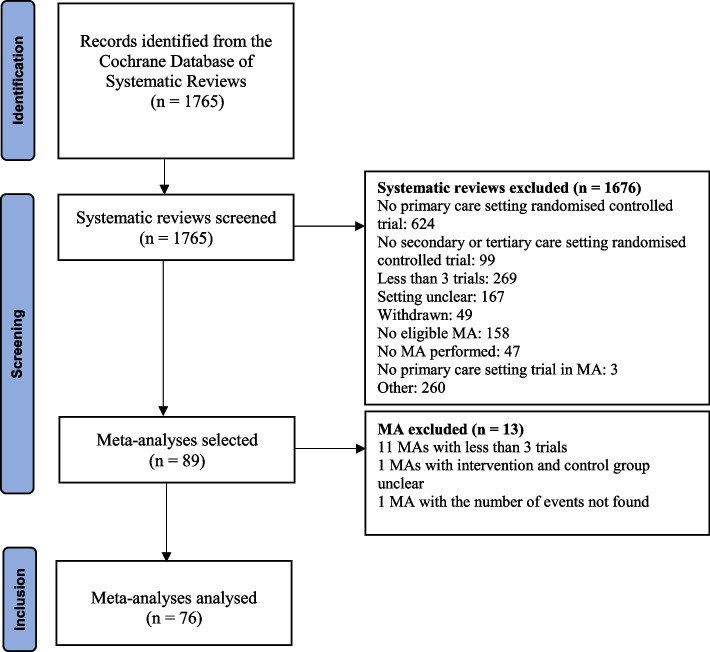
Flowchart of the selection process

From 1765 identified systematic reviews, we selected 76 MAs with 1685 trials, from which 614 trials were selected (Fig. 1, full references of the 76 MAs in Additional file 1 and 2). Of these 76 MAs, 33 (43.4%) evaluated a pharmacological intervention, 15 (19.7%) had an active control and 32 (42.1%) concerned a subjective outcome. The median number of trials included per MA was 5 (interquartile range [IQR] 3.8–10). MA characteristics are detailed in Table 1.

**Table 1:** Characteristics of the meta-analyses (MAs) included (*n* = 76)

MA characteristics	
Year of publication, median (IQR)	2016 (2012–2018)
**Medical fields, n (%)**	
Psychiatry/addictology	29 (38.2)
Pneumology	10 (13.2)
Ear nose and throat	5 (6.6)
Cardiology	4 (5.3)
Dermatology	4 (5.3)
Pediatrics	4 (5.3)
Infectious diseases	3 (3.9)
Others	17 (22.4)
**Intervention in experimental group, n (%)**	
Pharmacological	33 (43.4)
Non-pharmacological	43 (56.6)
**Intervention in control group, n (%)**	
Active comparator	15 (19.7)
Placebo	15 (19.7)
No added intervention (i.e., usual care)	34 (44.7)
Sham comparator	1 (1.3)
Other	11 (14.6)
**Outcome objectivity, n (%)**	
All-cause mortality	2 (2.6)
Objectively assessed	10 (13.2)
Objectively assessed but influenced by clinician or patient	31 (40.8)
Subjectively assessed	32 (42.1)
Unclear	1 (1.3)
**Number of trials, median (IQR) (range)**	
Included	5 (3.8–10) (3 to 32)
PC-RCTs	2 (1–3) (1 to 17)
ST-RCTs	3 (1.8–6) (1 to 30)

No eligible MA refers to meta-analyses with several reasons for not selecting it: fewer than 3 trials, without both primary and secondary or tertiary care settings or without a primary efficacy outcome.

MA: meta-analysis.

MA: meta-analysis; IQR: interquartile range; PC-RCTs: primary care setting randomised controlled trials; ST-RCTs: secondary or tertiary care setting randomised controlled trials.

### Characteristics of selected trials

Among 614 trials selected, 230 were PC-RCTs and 384 ST-RCTs (Table 2). The median sample size was 276 (IQR 146–561.5) for PC-RCTs and 139 (IQR 66–284.3) for ST-RCTs. The median number of centers was 13 (IQR 3–40.5) and 1 (IQR 1–3), respectively. A total of 61 (62.9%) PC-RCTs were at low risk of bias for blinding of outcome assessors as compared with 83 (43.4%) ST-RCTs. More characteristics of the PC-RCTs included in this study are available in Additional file 3.

**Table 2:** Characteristics of the included trials

Trial characteristics	PC-RCTs	ST-RCTs
	***N*** **= 230**	***N*** **= 384**
**Year of publication, median (IQR)**	2003 (1994–2010)	2005 (1996–2011)
**Sample size, median (IQR)**	276 (146–561.5)	139 (166–284.3)
**Number of centers, median (IQR)**	13 (3–40.5)	1 (1–3)
**Medical fields**		
Psychiatry/Addictology	109 (47.4)	184 (47.9)
Pneumology	15 (6.5)	66 (17.2)
Ear nose and throat	12 (5.2)	12 (3.1)
Cardiology	9 (3.9)	15 (3.9)
Dermatology	6 (2.6)	11 (2.9)
Pediatrics	11 (4.8)	21 (5.5)
Infectious diseases	8 (3.5)	4 (1.1)
Others	60 (26.1)	71 (18.5)
**Intervention in experimental group, n (%)**		
Pharmacological	79 (34.3)	171 (44.5)
Non-pharmacological	151 (65.7)	213 (55.5)
**Intervention in control group, n (%)**		
Active comparator	35 (15.2)	78 (20.3)
Placebo	29 (12.6)	39 (10.2)
No added intervention (i.e., usual care)	115 (50.0)	188 (49.0)
Sham comparator	2 (0.9)	2 (0.5)
Other	49 (21.3)	77 (20.0)
**Outcome objectivity, n (%)**		
All-cause mortality	3 (1.3)	32 (8.3)
Objectively assessed	55 (23.9)	48 (12.5)
Objectively assessed but influenced by clinician or patient	95 (41.3)	170 (44.3)
Subjectively assessed	75 (32.6)	132 (34.4)
Unclear	2 (0.9)	2 (0.5)
**Cochrane Risk of Bias tool, n (%)**		
Random sequence generation		
Low	120 (57.4)	197 (57.4)
High	11 (5.3)	11 (3.2)
Unclear	78 (37.3)	135 (39.4)
Allocation concealment		
Low	116 (50.4)	165 (43.0)
High	21 (9.1)	26 (6.8)
Unclear	93 (40.4)	193 (50.3)
Blinding of participants and personnel		
Low	68 (45.6)	74 (30.1)
High	33 (22.2)	62 (25.2)
Unclear	48 (32,2)	110 (44.7)
Blinding of outcome assessors		
Low	61 (62.9)	83 (43.4)
High	14 (14.4)	50 (26.2)
Unclear	22 (22.7)	58 (30.4)
Incomplete data outcome		
Low	135 (64.3)	215 (62.7)
High	34 (16.2)	53 (15.4)
Unclear	41 (19.5)	75 (21.9)
Selective reporting		
Low	85 (63.9)	144 (58.5)
High	9 (6.8)	23 (9.4)
Unclear	39 (29.3)	79 (32.1)

IQR: interquartile range, PC-RCT: primary care setting randomised controlled trial, ST-RCT: secondary or tertiary care setting randomised controlled trial.

### Differences in intervention effect estimates between PC-RCTs and ST-RCTs

**Fig. 2:**
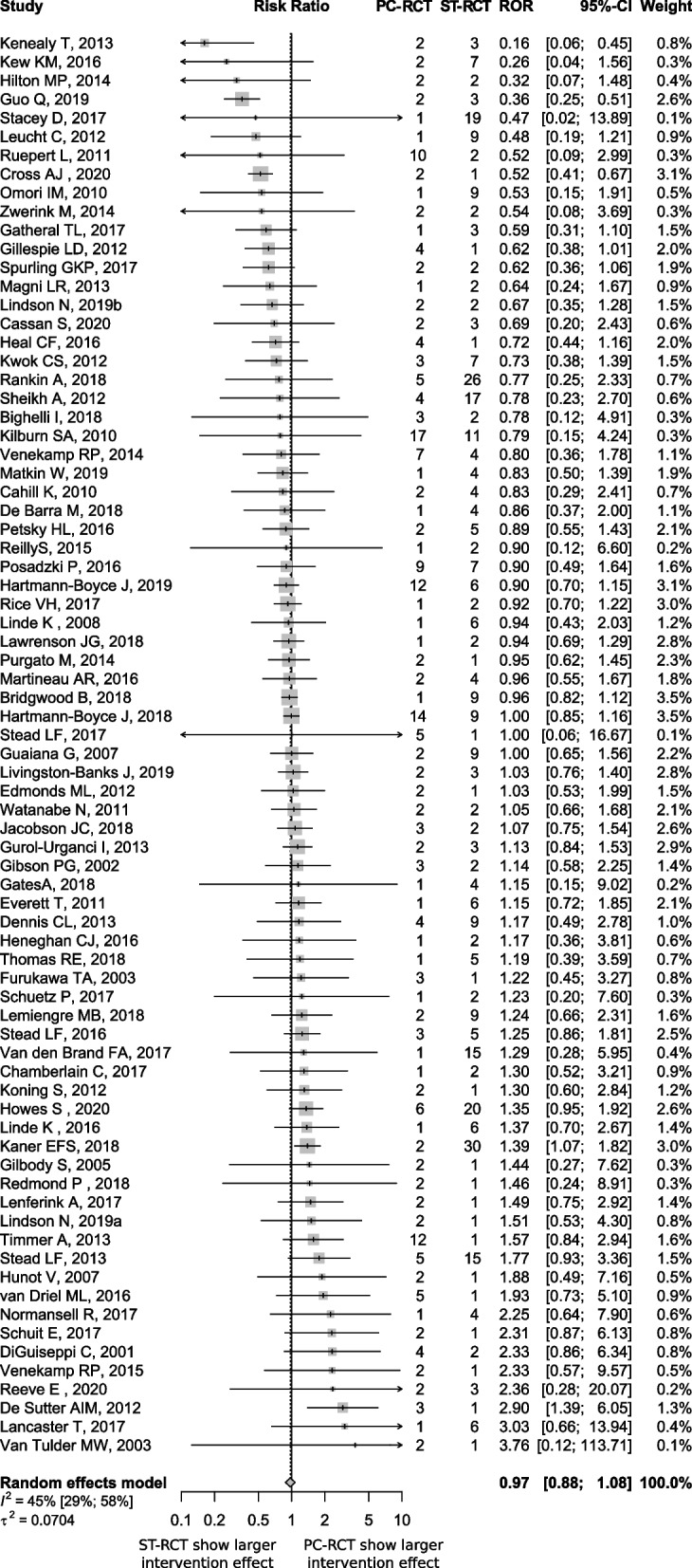
Difference in the intervention effect estimates between randomised controlled trials in primary care versus secondary or tertiary settings

We found no statistical difference in intervention effect estimates between PC-RCTs and ST-RCTs; the combined ROR was 0.97 (95% CI 0.88 to 1.08) (Fig. 2). The heterogeneity across MAs was moderate (I^2^ = 45, 95%CI 29 to 58%, between–meta-analysis variance τ^2^ = 0.07).

PC-RCTs: primary care setting randomised controlled trial.

ST-RCTs: secondary or tertiary care setting randomised controlled trial.

#### Subgroup analyses

**Fig. 3:**
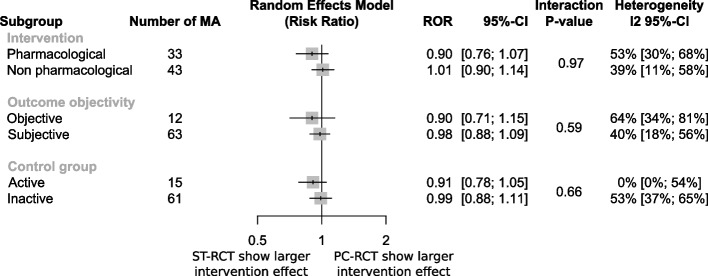
Difference in intervention effect estimates for the subgroup analyses

ROR: ratio of odds ratio; CI: confidence interval; MA: meta-analysis; PC-RCTs: primary care setting randomised controlled trials; ST-RCTs: secondary or tertiary care setting randomised controlled trials.

For the 33 MAs assessing pharmacological interventions, the combined ROR was estimated at 0.90 (95% CI 0.76 to 1.07), and for the 43 MAs assessing non-pharmacological interventions, the combined ROR was 1.01 (95% CI 0.90 to 1.14). The interaction test was not statistically significant (*P* = 0.97). We did not find a significant interaction in the other subgroup analyses (Fig. 3).

#### Sensitivity analyses

**Fig. 4:**
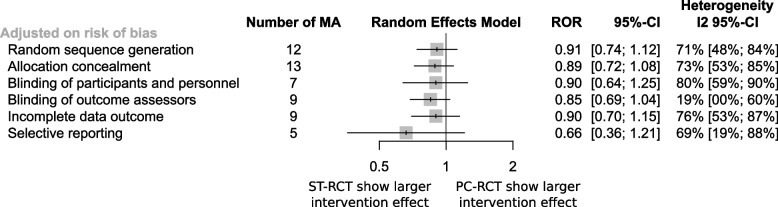
Difference in intervention effect estimates when adjusting on risk of bias

ROR: ratio of odds ratio; CI: confidence interval; MA: meta-analysis; PC-RCTs: primary care setting randomised controlled trials; ST-RCTs: secondary or tertiary care setting randomised controlled trials.

Results were consistent when adjusting on each item of the risk of bias (Fig. 4).

## Discussion

In this meta-epidemiological study of 76 MAs, we did not observe a significant difference in intervention effect estimates between randomised controlled trials conducted in primary care versus secondary or tertiary care settings. Consistent results were found in subgroup and sensitivity analyses. Our result and the way we worked to obtain it satisfy the 10 criteria as treated by Moustgaard et al. [ 17].

To our knowledge, our meta-epidemiological study is the first to compare intervention effect estimates between PC-RCTs and ST-RCTs. The number of MAs analysed is the major strength of this study. In 2016, Dechartres et al. found that the median number of MAs per meta-epidemiological study was 29 versus 76 MAs in our study [18]. Nevertheless, we restricted our study to Cochrane reviews, which could limit the generalizability of our results. Cochrane reviews were shown to have better methodological quality [19] and to be better reported [20] than non-Cochrane reviews. We performed an analysis of MAs of binary outcomes, so our results cannot be extrapolated to MAs with continuous outcomes. MAs with continuous outcomes have more subjective outcomes, are less frequently blinded [21]and have higher heterogeneity [22] than those with binary outcomes. Finally, another limitation may be the definition of primary care settings. Primary care is often difficult and poorly defined in the literature and can include a large range of professions and professionals [14]. In Cochrane reviews, the description of the type of healthcare professionals in charge of including, following up and assessing patients was seldom reported. This omission could have led us to misclassify some trials or also to exclude some trials because of uncertainty. Nevertheless, the selection and the extraction process were performed in duplicate by health professionals (i.e., a pharmacy resident, a general medicine resident and a professor of general medicine in case of discrepancy), which helped limit the uncertainty of some terms and reduced the risk of errors.

Our result does not support GPs’ general feeling that results from PC-RCTs differ from those derived from ST-RCTs [3, 5]. Our study is sufficiently powered, and the ROR point estimate was very close to 1, which indeed supports the conclusion of an absence of differences in intervention effect estimates between PC-RCTs and ST-RCTs. However, among the 76 MAs, 29 (38.2%) were related to psychiatry (mainly depression) or addictology (mainly tobacco use) and 10 (13.2%) to pneumology. Very few MAs were dedicated to cardiovascular conditions (*n* = 4, 5.3%) or diabetes (*n* = 1, 1.3%). So the medical fields represented in our study do not well match those mostly frequently encountered in primary care, typically cardiovascular diseases (13.6%), muskuloskeletal troubles (12.6%) and pneumology (12.5%), with psychatric diseases representing only 8.5% of the consultations [23]. Therefore, how we selected the MAs for our study led to a sample of diseases that does not perfectly reflect the diseases mainly managed in primary care. Other studies should probably focus on specific medical fields or diseases rather than considering all MAs, whatever the medical field.

Another point is that we focused on binary outcomes, but in cardiovascular diseases or diabetes, most trials use surrogate or intermediate endpoints, which are mainly continuous ones, such as cholesterol level, blood pressure or HbA1c level [24, 25]. This situation may also have led to discarding some MAs focused on diseases that are highly prevalent in primary care.

Finally, although the present study focused on the settings, intervention effect estimates also depend on the target population, defined by selection criteria and/or the type/intensity of the intervention assessed. Due to the limited size of our study, no adjustment on these characteristics could be done while analyzing data. Therefore, one of the remaining questions, which is beyond the scope of the present work, is whether patients included in PC-RCTs are similar to patients included in ST-RCTs.

## Conclusion

We found no significant difference in intervention effect estimates between PC-RCTs and ST-RCTs. Nevertheless, the main medical fields selected are not fully representative of primary care consultations. Further studies focusing on medical fields highly prevalent in primary care are needed.

## Supplementary Information

**Additional file 1. **List of included meta-analyses (*n* = 76).

**Additional file 2.** Description of the 76 meta-analyses.

**Additional file 3.** Characteristics of the primary care setting randomised controlled trials included.

## Data Availability

The datasets used and/or analysed during the current study are available in the *Additional file.*
