# Supplementary material for: Intervention effect estimates in randomised controlled trials conducted in primary care versus secondary or tertiary care settings: a meta-epidemiological study
Source: BMC Med Res Methodol. 2022 Dec 22;22:329. doi: 10.1186/s12874-022-01815-2 (PMC9773496; doi:10.1186/s12874-022-01815-2)
Supplement: Supplementary file 1 — Additional file 1. List of included meta-analyses (n = 76). [file 12874_2022_1815_MOESM1_ESM.docx]

## **Additional File 1.** List of included meta-analyses (n=76)

1. Cross AJ, Elliott, RA, Petrie, K, Kuruvilla, L, George J. Interventions for improving medication‐taking ability and adherence in older adults prescribed multiple medications. *Cochrane Database of Systematic Review* *2020*, Issue 5. Art. No.: CD012419. DOI: 10.1002/14651858.CD012419.pub2.

2. Cassan S, Thompson, MJ, Perera, R, Glasziou, PP, Del Mar, CB, Heneghan, CJ, Hayward G. Corticosteroids as standalone or add‐on treatment for sore throat. *Cochrane Database of Systematic Review* *2020*.  Issue 5. Art. No.: CD008268. DOI: 10.1002/14651858.CD008268.pub3.

3. Reeve E, Jordan V, Thompson W, Sawan M, Todd A, Gammie TM, et al. Withdrawal of antihypertensive drugs in older people. *Cochrane Database of Systematic Reviews 2020,* Issue 6. Art. No.: CD012572. DOI: 10.1002/14651858.CD012572.pub2.

4. Howes S, Hartmann‐Boyce, J, Livingstone‐Banks, J, Hong, B, Lindson N. Antidepressants for smoking cessation*. Cochrane Database of Systematic Reviews 2020,* Issue 4. Art. No.: CD000031. DOI: 10.1002/14651858.CD000031.pub5.

5. Matkin W, Ordóñez‐Mena, JM, Hartmann‐Boyce J. Telephone counselling for smoking cessation. *Cochrane Database of Systematic Reviews 2019,* Issue 5. Art. No.: CD002850. DOI: 10.1002/14651858.CD002850.pub4.

6. Livingstone‐Banks J, Ordóñez‐Mena, JM, Hartmann‐Boyce J. Print‐based self‐help interventions for smoking cessation. *Cochrane Database of Systematic Reviews 2019,* Issue 1. Art. No.: CD001118. DOI: 10.1002/14651858.CD001118.pub4.

7. Lindson N, Thompson, TP, Ferrey, A, Lambert, JD, Aveyard P. Motivational interviewing for smoking cessation. *Cochrane Database of Systematic Reviews 2019*, Issue 7. Art. No.: CD006936. DOI: 10.1002/14651858.CD006936.pub4.

8. Lindson N, Klemperer, E, Hong, B, Ordóñez‐Mena, JM, Aveyard P. Smoking reduction interventions for smoking cessation. *Cochrane Database of Systematic Reviews 2019,* Issue 9. Art. No.: CD013183. DOI: 10.1002/14651858.CD013183.pub2.

9. Hartmann‐Boyce J, Hong, B, Livingstone‐Banks, J, Wheat, H, Fanshawe T. Additional behavioural support as an adjunct to pharmacotherapy for smoking cessation. *Cochrane Database of Systematic Reviews 2019,* Issue 6. Art. No.: CD009670. DOI: 10.1002/14651858.CD009670.pub4.

10. Guo Q, Goldenberg, JZ, Humphrey, C, El Dib, R, Johnston B. Probiotics for the prevention of pediatric antibiotic‐associated diarrhea. *Cochrane Database of Systematic Reviews 2019,* Issue 4. Art. No.: CD004827. DOI: 10.1002/14651858.CD004827.pub5.

11. Kaner EFS, Beyer, FR, Muirhead, C, Campbell, F, Pienaar, ED, Bertholet, N, Daeppen, JB, Saunders, JB, Burnand B. Effectiveness of brief alcohol interventions in primary care populations. *Cochrane Database of Systematic Reviews 2018,* Issue 2. Art. No.: CD004148. DOI: 10.1002/14651858.CD004148.pub4.

12. de Barra M, Scott, CL, Scott, NW, Johnston, M, de Bruin, M, Nkansah, N, Bond, CM, Matheson, CI, Rackow, P, Williams, AJ, Watson M. Pharmacist services for non‐hospitalised patients. *Cochrane Database of Systematic Reviews 2018,* Issue 9. Art. No.: CD013102. DOI: 10.1002/14651858.CD013102.

13. Rankin A, Cadogan, CA, Patterson, SM, Kerse, N, Cardwell, CR, Bradley, MC, Ryan, C, Hughes C. Interventions to improve the appropriate use of polypharmacy for older people. *Cochrane Database of Systematic Reviews 2018,* Issue 9. Art. No.: CD008165. DOI: 10.1002/14651858.CD008165.pub4.

14. Thomas RE, Lorenzetti D. Interventions to increase influenza vaccination rates of those 60 years and older in the community. *Cochrane Database of Systematic Reviews 2018,* Issue 5. Art. No.: CD005188. DOI: 10.1002/14651858.CD005188.pub4.

15. Gates A, Gates, M, Vandermeer, B, Johnson, C, Hartling, L, Johnson, DW, Klassen T. Glucocorticoids for croup in children. *Cochrane Database of Systematic Reviews 2018,* Issue 8. Art. No.: CD001955. DOI: 10.1002/14651858.CD001955.pub4.

16. Lemiengre MB, van Driel, ML, Merenstein, D, Liira, H, Mäkelä, M, De Sutter A. Antibiotics for acute rhinosinusitis in adults. *Cochrane Database of Systematic Reviews 2018*, Issue 9. Art. No.: CD006089. DOI: 10.1002/14651858.CD006089.pub5.

17. Lawrenson JG, Graham‐Rowe, E, Lorencatto, F, Burr, J, Bunce, C, Francis, JJ, Aluko, P, Rice, S, Vale, L, Peto, T, Presseau, J, Ivers, N, Grimshaw J. Interventions to increase attendance for diabetic retinopathy screening. *Cochrane Database of Systematic Reviews 2018,* Issue 1. Art. No.: CD012054. DOI: 10.1002/14651858.CD012054.pub2.

18. Redmond P, Grimes, TC, McDonnell, R, Boland, F, Hughes, C, Fahey T. Impact of medication reconciliation for improving transitions of care. *Cochrane Database of Systematic Reviews 2018,* Issue 8. Art. No.: CD010791. DOI: 10.1002/14651858.CD010791.pub2.

19. Jacobson Vann JC, Jacobson, RM, Coyne‐Beasley, T, Asafu‐Adjei, JK, Szilagyi P. Patient reminder and recall interventions to improve immunization rates. *Cochrane Database of Systematic Reviews 2018,* Issue 1. Art. No.: CD003941. DOI: 10.1002/14651858.CD003941.pub3.

20. Bridgwood B, Lager, KE, Mistri, AK, Khunti, K, Wilson, AD, Modi P. Interventions for improving modifiable risk factor control in the secondary prevention of stroke. *Cochrane Database of Systematic Reviews 2018,* Issue 5. Art. No.: CD009103. DOI: 10.1002/14651858.CD009103.pub3.

21. Hartmann‐Boyce J, Chepkin, SC, Ye, W, Bullen, C, Lancaster T. Nicotine replacement therapy versus control for smoking cessation. *Cochrane Database of Systematic Reviews 2018,* Issue 5. Art. No.: CD000146. DOI: 10.1002/14651858.CD000146.pub5.

22. Bighelli I, Castellazzi, M, Cipriani, A, Girlanda, F, Guaiana, G, Koesters, M, Turrini, G, Furukawa, TA, Barbui C. Antidepressants versus placebo for panic disorder in adults. *Cochrane Database of Systematic Reviews 2018,* Issue 4. Art. No.: CD010676. DOI: 10.1002/14651858.CD010676.pub2.

23. van den Brand FA, Nagelhout, GE, Reda, AA, Winkens, B, Evers, SMAA, Kotz, D, van Schayck O. Healthcare financing systems for increasing the use of tobacco dependence treatment. *Cochrane Database of Systematic Reviews 2017*, Issue 9. Art. No.: CD004305. DOI: 10.1002/14651858.CD004305.pub5.

24. Rice VH, Heath, L, Livingstone‐Banks, J, Hartmann‐Boyce J. Nursing interventions for smoking cessation. *Cochrane Database of Systematic Reviews 2017,* Issue 12. Art. No.: CD001188. DOI: 10.1002/14651858.CD001188.pub5.

25. Spurling GKP, Del Mar, CB, Dooley, L, Foxlee, R, Farley R. Delayed antibiotic prescriptions for respiratory infections. *Cochrane Database of Systematic Reviews 2017*, Issue 9. Art. No.: CD004417. DOI: 10.1002/14651858.CD004417.pub5.

26. Lenferink A, Brusse‐Keizer, M, van der Valk, PDLPM, Frith, PA, Zwerink, M, Monninkhof, EM, van der Palen, J, Effing T. Self‐management interventions including action plans for exacerbations versus usual care in patients with chronic obstructive pulmonary disease. *Cochrane Database of Systematic Reviews 2017*, Issue 8. Art. No.: CD011682. DOI: 10.1002/14651858.CD011682.pub2.

27. Lancaster T, Stead L. Individual behavioural counselling for smoking cessation. *Cochrane Database of Systematic Reviews 2017,* Issue 3. Art. No.: CD001292. DOI: 10.1002/14651858.CD001292.pub3.

28. Stead LF, Carroll, AJ, Lancaster T. Group behaviour therapy programmes for smoking cessation. *Cochrane Database of Systematic Reviews 2017,* Issue 3. Art. No.: CD001007. DOI: 10.1002/14651858.CD001007.pub3.

29. Schuetz P, Wirz, Y, Sager, R, Christ‐Crain, M, Stolz, D, Tamm, M, Bouadma, L, Luyt, CE, Wolff, M, Chastre, J, Tubach, F, Kristoffersen, KB, Burkhardt, O, Welte, T, Schroeder, S, Nobre, V, Wei, L, Bucher, HCC, Bhatnagar, N, Annane, D, Reinhart, K, Branche, A, Damas, P, Nijsten, M, de Lange, DW, Deliberato, RO, Lima, SSS, Maravić‐Stojković, V, Verduri, A, Cao, B, Shehabi, Y, Beishuizen, A, Jensen, JUS, Corti, C, Van Oers, JA, Falsey, AR, de Jong, E, Oliveira, CF, Beghe, B, Briel, M, Mueller B. Procalcitonin to initiate or discontinue antibiotics in acute respiratory tract infections. *Cochrane Database of Systematic Reviews 2017,* Issue 10. Art. No.: CD007498. DOI: 10.1002/14651858.CD007498.pub3.

30. Gatheral TL, Rushton, A, Evans, DJW, Mulvaney, CA, Halcovitch, NR, Whiteley, G, Eccles, FJR, Spencer S. Personalised asthma action plans for adults with asthma*. Cochrane Database of Systematic Reviews 2017,* Issue 4. Art. No.: CD011859. DOI: 10.1002/14651858.CD011859.pub2.

31. Normansell R, Kew, KM, Mathioudakis A. Interventions to improve inhaler technique for people with asthma. *Cochrane Database of Systematic Reviews 2017,* Issue 3. Art. No.: CD012286. DOI: 10.1002/14651858.CD012286.pub2*.*

32. Chamberlain C, O’Mara‐Eves, A, Porter, J, Coleman, T, Perlen, SM, Thomas, J, McKenzie J. Psychosocial interventions for supporting women to stop smoking in pregnancy. *Cochrane Database of Systematic Reviews 2017,* Issue 2. Art. No.: CD001055. DOI: 10.1002/14651858.CD001055.pub5.

33. Stacey D, Légaré, F, Lewis, K, Barry, MJ, Bennett, CL, Eden, KB, Holmes‐Rovner, M, Llewellyn‐Thomas, H, Lyddiatt, A, Thomson, R, Trevena L. Decision aids for people facing health treatment or screening decisions. *Cochrane Database of Systematic Reviews 2017,* Issue 4. Art. No.: CD001431. DOI: 10.1002/14651858.CD001431.pub5.

34. Schuit E, Panagiotou, OA, Munafò, MR, Bennett, DA, Bergen, AW, David S. Pharmacotherapy for smoking cessation: effects by subgroup defined by genetically informed biomarkers. *Cochrane Database of Systematic Reviews 2017,* Issue 9. Art. No.: CD011823. DOI: 10.1002/14651858.CD011823.pub2.

35. van Driel ML, Morledge, MD, Ulep, R, Shaffer, JP, Davies, P, Deichmann R. Interventions to improve adherence to lipid‐lowering medication. *Cochrane Database of Systematic Reviews 2016,* Issue 12. Art. No.: CD004371. DOI: 10.1002/14651858.CD004371.pub4.

36. Linde K, Allais, G, Brinkhaus, B, Fei, Y, Mehring, M, Shin, BC, Vickers, A, White A. Acupuncture for the prevention of tension‐type headache. *Cochrane Database of Systematic Reviews 2016,* Issue 4. Art. No.: CD007587. DOI: 10.1002/14651858.CD007587.pub2.

37. Heal CF, Banks, JL, Lepper, PD, Kontopantelis, E, van Driel M. Topical antibiotics for preventing surgical site infection in wounds healing by primary intention. *Cochrane Database of Systematic Reviews 2016,* Issue 4. Art. No.: CD007587. DOI: 10.1002/14651858.CD007587.pub2.

38. Heneghan CJ, Garcia‐Alamino, JM, Spencer, EA, Ward, AM, Perera, R, Bankhead, C, Alonso‐Coello, P, Fitzmaurice, D, Mahtani, KR, Onakpoya I. Self‐monitoring and self‐management of oral anticoagulation. *Cochrane Database of Systematic Reviews 2016*, Issue 11. Art. No.: CD011426. DOI: 10.1002/14651858.CD011426.pub2.

39. Stead LF, Koilpillai, P, Fanshawe, TR, Lancaster T. Combined pharmacotherapy and behavioural interventions for smoking cessation. *Cochrane Database of Systematic Reviews 2016,* Issue 3. Art. No.: CD008286. DOI: 10.1002/14651858.CD008286.pub3.

40. Posadzki P, Mastellos, N, Ryan, R, Gunn, LH, Felix, LM, Pappas, Y, Gagnon, MP, Julious, SA, Xiang, L, Oldenburg, B, Car J. Automated telephone communication systems for preventive healthcare and management of long‐term conditions. *Cochrane Database of Systematic Reviews 2016,* Issue 12. Art. No.: CD009921. DOI: 10.1002/14651858.CD009921.pub2.

41. Martineau AR, Cates, CJ, Urashima, M, Jensen, M, Griffiths, AP, Nurmatov, U, Sheikh, A, Griffiths C. Vitamin D for the management of asthma. *Cochrane Database of Systematic Reviews 2016,* Issue 9. Art. No.: CD011511. DOI: 10.1002/14651858.CD011511.pub2.

42. Petsky HL, Kew, KM, Chang A. Exhaled nitric oxide levels to guide treatment for children with asthma. *Cochrane Database of Systematic Reviews 2016,* Issue 11. Art. No.: CD011439. DOI: 10.1002/14651858.CD011439.pub2.

43. Kew KM, Cates C. Home telemonitoring and remote feedback between clinic visits for asthma. *Cochrane Database of Systematic Reviews 2016*, Issue 8. Art. No.: CD011714. DOI: 10.1002/14651858.CD011714.pub2.

44. Venekamp RP, Sanders, SL, Glasziou, PP, Del Mar, CB, Rovers M. Antibiotics for acute otitis media in children. *Cochrane Database of Systematic Reviews 2015*, Issue 6. Art. No.: CD000219. DOI: 10.1002/14651858.CD000219.pub4.

45. Reilly S, Miranda‐Castillo, C, Malouf, R, Hoe, J, Toot, S, Challis, D, Orrell M. Case management approaches to home support for people with dementia. *Cochrane Database of Systematic Reviews 2015,* Issue 1. Art. No.: CD008345. DOI: 10.1002/14651858.CD008345.pub2.

46. Hilton MP, Pinder D. The Epley (canalith repositioning) manoeuvre for benign paroxysmal positional vertigo. *Cochrane Database of Systematic Reviews 2014,* Issue 12. Art. No.: CD003162. DOI: 10.1002/14651858.CD003162.pub3.

47. Zwerink M, Brusse‐Keizer, M, van der Valk, PDLPM, Zielhuis, GA, Monninkhof, EM, van der Palen, J, Frith, PA, Effing T. Self management for patients with chronic obstructive pulmonary disease. *Cochrane Database of Systematic Reviews 2014,* Issue 3. Art. No.: CD002990. DOI: 10.1002/14651858.CD002990.pub3.

48. Purgato M, Papola, D, Gastaldon, C, Trespidi, C, Magni, LR, Rizzo, C, Furukawa, TA, Watanabe, N, Cipriani, A, Barbui C. Paroxetine versus other anti‐depressive agents for depression. *Cochrane Database of Systematic Reviews 2014,* Issue 4. Art. No.: CD006531. DOI: 10.1002/14651858.CD006531.pub2.

49. Venekamp RP, Thompson, MJ, Hayward, G, Heneghan, CJ, Del Mar, CB, Perera, R, Glasziou, PP, Rovers M. Systemic *corticosteroids for acute sinusitis. Cochrane Database of Systematic Reviews 2014, Issue 3. Art. No.: CD008115. DOI: 10.1002/14651858.CD008115.pub3.*

50. Stead LF, Buitrago, D, Preciado, N, Sanchez, G, Hartmann‐Boyce, J, Lancaster T. Physician advice for smoking cessation. *Cochrane Database of Systematic Reviews 2013*, Issue 5. Art. No.: CD000165. DOI: 10.1002/14651858.CD000165.pub4.

51. Kenealy T, Arroll B. Antibiotics for the common cold and acute purulent rhinitis. *Cochrane Database of Systematic Reviews 2013, Issue 6. Art. No.: CD000247. DOI: 10.1002/14651858.CD000247.pub3.*

52. Gurol‐Urganci I, de Jongh, T, Vodopivec‐Jamsek, V, Car, J, Atun R. Mobile phone messaging for communicating results of medical investigations. *Cochrane Database of Systematic Reviews 2012,* Issue 6. Art. No.: CD007456. DOI: 10.1002/14651858.CD007456.pub2.

53. Timmer A, Günther, J, Motschall, E, Rücker, G, Antes, G, Kern W. Pelargonium sidoides extract for treating acute respiratory tract infections. *Cochrane Database of Systematic Reviews 2013,* Issue 10. Art. No.: CD006323. DOI: 10.1002/14651858.CD006323.pub3.

54. Dennis CL, Dowswell T. Psychosocial and psychological interventions for preventing postpartum depression. *Cochrane Database of Systematic* Reviews 2012, Issue 6. Art. No.: CD007456. DOI: 10.1002/14651858.CD007456.pub2

55. Magni LR, Purgato, M, Gastaldon, C, Papola, D, Furukawa, TA, Cipriani, A, Barbui C. Fluoxetine versus other types of pharmacotherapy for depression. *Cochrane Database of Systematic Reviews 2013,* Issue 7. Art. No.: CD004185. DOI: 10.1002/14651858.CD004185.pub3

56. Koning S, van der Sande, R, Verhagen, AP, van Suijlekom‐Smit, LWA, Morris, AD, Butler, CC, Berger, M, van der Wouden J. Interventions for impetigo. *Cochrane Database of Systematic Reviews 2012,* Issue 1. Art. No.: CD003261. DOI: 10.1002/14651858.CD003261.pub3.

57. Gillespie LD, Robertson, MC, Gillespie, WJ, Sherrington, C, Gates, S, Clemson, LM, Lamb S. Interventions for preventing falls in older people living in the community. *Cochrane Database of Systematic Reviews 2012,* Issue 9. Art. No.: CD007146. DOI: 10.1002/14651858.CD007146.pub3.

58. Sheikh A, Hurwitz, B, van Schayck, CP, McLean, S, Nurmatov U. Antibiotics versus placebo for acute bacterial conjunctivitis. *Cochrane Database of Systematic Reviews 2012,* Issue 9. Art. No.: CD001211. DOI: 10.1002/14651858.CD001211.pub3.

59. De Sutter AIM, van Driel, ML, Kumar, AA, Lesslar, O, Skrt A. Oral antihistamine‐decongestant‐analgesic combinations for the common cold. *Cochrane Database of Systematic Reviews 2012,* Issue 2. Art. No.: CD004976. DOI: 10.1002/14651858.CD004976.pub3.

60. Leucht C, Huhn, M, Leucht S. Amitriptyline versus placebo for major depressive disorder. *Cochrane Database of Systematic Reviews 2012,* Issue 12. Art. No.: CD009138. DOI: 10.1002/14651858.CD009138.pub2.

61. Kwok CS, Gibbs, S, Bennett, C, Holland, R, Abbott R. Topical treatments for cutaneous warts. *Cochrane Database of Systematic Reviews 2012,* Issue 9. Art. No.: CD001781. DOI: 10.1002/14651858.CD001781.pub3.

62. Edmonds ML, Milan, SJ, Brenner, BE, Camargo Jr, CA, Rowe B. Inhaled steroids for acute asthma following emergency department discharge. *Cochrane Database of Systematic Reviews 2012,* Issue 12. Art. No.: CD002316. DOI: 10.1002/14651858.CD002316.pub2.

63. Ruepert L, Quartero, AO, de Wit, NJ, van der Heijden, GJ, Rubin, G, Muris J. Bulking agents, antispasmodics and antidepressants for the treatment of irritable bowel syndrome. *Cochrane Database of Systematic Reviews 2011,* Issue 8. Art. No.: CD003460. DOI: 10.1002/14651858.CD003460.pub3.

64. Watanabe N, Omori, IM, Nakagawa, A, Cipriani, A, Barbui, C, Churchill, R, Furukawa T. Mirtazapine versus other antidepressive agents for depression. *Cochrane Database of Systematic Reviews 2011,* Issue 12. Art. No.: CD006528. DOI: 10.1002/14651858.CD006528.pub2.

65. Everett T, Bryant, A, Griffin, MF, Martin‐Hirsch, PPL, Forbes, CA, Jepson R. Interventions targeted at women to encourage the uptake of cervical screening. *Cochrane Database of Systematic Reviews 2021,* Issue 9. Art. No.: CD002834. DOI: 10.1002/14651858.CD002834.pub3.

66. Cahill K, Lancaster, T, Green N. Stage‐based interventions for smoking cessation. *Cochrane Database of Systematic Reviews 2010,* Issue 11. Art. No.: CD004492. DOI: 10.1002/14651858.CD004492.pub4.

67. Omori IM, Watanabe, N, Nakagawa, A, Cipriani, A, Barbui, C, McGuire, H, Churchill, R, Furukawa T. Fluvoxamine versus other anti‐depressive agents for depression. *Cochrane Database of Systematic Reviews 2010,* Issue 3. Art. No.: CD006114. DOI: 10.1002/14651858.CD006114.pub2.

68. Kilburn SA, Featherstone, P, Higgins, B, Brindle R. Interventions for cellulitis and erysipelas. *Cochrane Database of Systematic Reviews 2010,* Issue 6. Art. No.: CD004299. DOI: 10.1002/14651858.CD004299.pub2.

69. Linde K, Berner, MM, Kriston L. St John’s wort for major depression. *Cochrane Database of Systematic Reviews 2008,* Issue 4. Art. No.: CD000448. DOI: 10.1002/14651858.CD000448.pub3.

70. Hunot V, Churchill, R, Teixeira, V, Silva de Lima M. Psychological therapies for generalised anxiety disorder. *Cochrane Database of Systematic Reviews 2007,* Issue 1. Art. No.: CD001848. DOI: 10.1002/14651858.CD001848.pub4

71. Guaiana G, Barbui, C, Hotopf M. Amitriptyline for depression. *Cochrane Database of Systematic Reviews 2007,* Issue 3. Art. No.: CD004186. DOI: 10.1002/14651858.CD004186.pub2.

72. Gilbody S, House, A, Sheldon T. Screening and case finding instruments for depression. *Cochrane Database of Systematic Reviews 2005,* Issue 4. Art. No.: CD002792. DOI: 10.1002/14651858.CD002792.pub2.

73. Furukawa TA, McGuire, H, Barbui C. Low dosage tricyclic antidepressants for depression. *Cochrane Database of Systematic Reviews 2003,* Issue 3. Art. No.: CD003197. DOI: 10.1002/14651858.CD003197.

74. van Tulder MW, Touray, T, Furlan, AD, Solway, S, Bouter L. Muscle relaxants for non‐specific low‐back pain. *Cochrane Database of Systematic Reviews 2003,* Issue 2. Art. No.: CD004252. DOI: 10.1002/14651858.CD004252.

75. Gibson PG, Powell, H, Wilson, A, Abramson, MJ, Haywood, P, Bauman, A, Hensley, MJ, Walters, EH, Roberts J. Self‐management education and regular practitioner review for adults with asthma. *Cochrane Database of Systematic Reviews 2002,* Issue 3. Art. No.: CD001117. DOI: 10.1002/14651858.CD001117.

76. DiGuiseppi C, Goss, CW, Higgins J. Interventions for promoting smoke alarm ownership and function. *Cochrane Database of Systematic Reviews 2001,* Issue 2. Art. No.: CD002246. DOI: 10.1002/14651858.CD002246.
