# Supplementary material for: Intervention effect estimates in randomised controlled trials conducted in primary care versus secondary or tertiary care settings: a meta-epidemiological study
Source: BMC Med Res Methodol. 2022 Dec 22;22:329. doi: 10.1186/s12874-022-01815-2 (PMC9773496; doi:10.1186/s12874-022-01815-2)
Supplement: Supplementary file 2 — Additional file 2. Description of the 76 meta-analyses. [file 12874_2022_1815_MOESM2_ESM.docx]

**Additional File 2.** Description of the 76 meta-analyses

| Name | Condition studied | Intervention in experimental arm | Intervention in the control arm | Outcome | Number of patients Total, median [min-max] | Combined OR [95% CI] | Heterogeneity  I^2^ (%) |
| --- | --- | --- | --- | --- | --- | --- | --- |
| Cross AJ, 2020 | Prescribed multiple medication | Mixed educational and behavioral interventions | No added intervention i.e. “usual care” | Adherence | 2517,  120  (4-1340) | 0.48  [0.31;0.75] | 71 |
| Cassan S, 2020 | Sore throat | Corticosteroids | Placebo | Complete resolution of pain at 24h | 851,  77  (57-565) | 0.3 [0.13;0.7] | 67 |
| Reeve E, 2020 | Hypertensive drugs withdrawal | Discontinuation treatment | No added intervention i.e. “usual care” | All cause of mortality | 568,  106  (77-385) | 2.6  [0.86;7.83] | 0 |
| Howes S, 2020 | Smoking cessation | Bupropion | Placebo/no pharmacy control | Smoking cessation | 1785,  251  (47-593) | 0.711.64  [0.53;0.94] | 20 |
| Matkin W, 2019 | Smoking cessation | Telephone counselling | Active comparator | Cessation at longest follow-up | 5930,  290  (40 – 1368) | 0.7  [0.51;0.98] | 65 |
| Livingston-Banks J, 2019 | Smoking cessation | No contact/face to face/ advice | No added intervention i.e. “usual care” | Long term abstinence | 6651,  1253  (397-2465) | 0.9  [0.75;1.09] | 0 |
| Lindson N, 2019 | Smoking cessation | Motivational intervention | Active comparator | Cessation | 1304,  210  (85-536) | 0.95  [0.55;1.65] | 73 |
| Lindson N, 2019 | Smoking cessation | Reduction to quit | Active comparator | Abstinence | 1376,  185  (70-697) | 0.54  [0.26;1.11] | 54 |
| Hartmann‐Boyce J, 2019 | Smoking cessation | Increasing behavioural support | Active comparator | Abstinence at longest follow up | 4586,  376  (64-925) | 0.97 [0.82;1.14] | 0 |
| Guo Q, 2019 | Pediatric antibiotic-associated diarrhea | Probiotics | Active control/placebo/no treatment | Incidence of diarrhea | 4181  112  (15–616) | 0.35  [0.24;0.51] | 58 |
| Kaner EFS, 2018 | Brief alcohol intervention | Brief intervention | Minimal/no intervention | Heavy drinkers at 12 months | 7623  366,5  (64-907) | 0.68  [0.55;0.83] | 73 |
| de Barra M, 2018 | Pharmacist services | Pharmacist intervention | No added intervention i.e. “usual care” | % outside blood pressure range | 3069  228  (55-730) | 0.39  [0.26;0.6] | 85 |
| Rankin A, 2018 | Polypharmacy for older people | Pharmaceutical care | No added intervention i.e. “usual care” | The proportion of patients with one or more potentially inappropriate medications | 2347  265,5  (22-697) | 0.6  [0.31;1.19] | 90 |
| Thomas RE, 2018 | Vaccination in older people | Reminder and recall | No added intervention i.e. “usual care” | Uptake of vaccination against influenza | \| 2639 \| \| --- \| \| 301 \| \| (95-900) \| \|  \| | 0.82  [0.51 ;1.32] | 83 |
| Gates A, 2018 | Croup in children | DXM | Active comparator | Return visits or (re)admissions or both by inpatient/outpatient | \| 306 \| \| --- \| \| 87 \| \| (86-133) \| \|  \| | 0.32  [0.14;0.74] | 12 |
| Lemiengre MB, 2018 | Rhinosinusitis in adults | Antibiotics | Placebo | Cure | \| 1306 \| \| --- \| \| 197,5 \| \| (127-384) \| \|  \| | 0.78  [0.57;1.08] | 44 |
| Lawrenson JG, 2018 | Diabetic retinopathy screening | Any QI intervention | No added intervention i.e. “usual care” | Proportion of participants attending screening | \| 200902 \| \| --- \| \| 336 \| \| (72-188169) \| \|  \| | 0.51  [0.41;0.64] | 91 |
| Redmond P, 2018 | Medication reconciliation for improving transition of care | Medication reconciliation | No added intervention i.e. “usual care” | At least 1 medication discrepancy per participant | \| 4312 \| \| --- \| \| 246 \| \| (29-851) \| \|  \| | 0.23  [0.12;0.43] | 93 |
| Jacobson Vann JC, 2018 | Recall intervention to improve vaccination | Patient reminder | No added intervention i.e. “usual care” | Immunized | \| 16176 \| \| --- \| \| 432 \| \| (112-6106) \| \|  \| | 0.52  [0.41;0.65] | 84 |
| Bridgwood B, 2018 | Stroke prevention | Organisational intervention | No added intervention i.e. “usual care” | Blood pressure target achievement | \| 19554 \| \| --- \| \| 303 \| \| (36-17511) \| \|  \| | 0.83  [0.79;0.88] | 0 |
| Hartmann‐Boyce J, 2018 | Smoking cessation | NRT | Placebo/no NRT | Smoking cessation | \| 14582 \| \| --- \| \| 295 \| \| (81-1843) \| \|  \| | 0.67  [0.6;0.75] | 0 |
| Bighelli I, 2018 | Panic disorder in adults | SSRIs (antidepressant) | Placebo | Failure to respond | \| 357 \| \| --- \| \| 56 \| \| (29-169) \| \|  \| | 0.25  [0.08;0.75] | 74 |
| van den Brand FA, 2017 | Smoking cessation | Full coverage | No added intervention i.e. “usual care” | Abstinence from smoking | \| 338 \| \| --- \| \| 70 \| \| (48-220) \| \|  \| | 0.29  [0.11;0.79] | 0 |
| Rice VH, 2017 | Smoking cessation | All nursing intervention | No added intervention i.e. “usual care” | Smoking cessation at longest follow-up | \| 13486 \| \| --- \| \| 280,5 \| \| (25-1942) \| \|  \| | 0.71  [0.61;0.82] | 38 |
| Spurling GKP, 2017 | Respiratory infection | Delayed antibiotic | Active comparator | Number of participants with pain on days 3 to 6 | \| 596 \| \| --- \| \| 212 \| \| (119-265) \| \|  \| | 1.28  [0.77 ;2.13] | 39 |
| Lenferink A, 2017 | COPD | Self-management | No added intervention i.e. “usual care” | Respiratory-related hospital admissions (number of patients with at least one admission) | \| 2564 \| \| --- \| \| 172 \| \| (38-743) \| \|  \| | 0.71 [0.51;098] | 51 |
| Lancaster T, 2017 | Smoking cessation | Counselling | Minimal contact control | Smoking cessation at longest follow-up | \| 2185 \| \| --- \| \| 163 \| \| (39-615) \| \|  \| | 0.62  [0.47;0.83] | 0 |
| Stead LF, 2017 | Smoking cessation | Group | Active comparator | Smoking cessation | \| 1379 \| \| --- \| \| 273 \| \| (64-1042) \| \|  \| | 0.83  [0.5;1.37] | 0 |
| Schuetz P, 2017 | Acute respiratory tract infections | Procalcitonin algorithm | No added intervention i.e. “usual care” | Mortality | \| 10046 \| \| --- \| \| 185,5 \| \| (27-1546) \| \|  \| | 0.89  [0.78;1.01] | 0 |
| Gatheral TL, 2017 | Asthma | PAAP | No added intervention i.e. “usual care” | Number of participants reporting at least 1 exacerbation requiring emergency department visit or hospitalisation. | \| 1260 \| \| --- \| \| 140 \| \| (74-906) \| \|  \| | 0.72  [0.47;1.11] | 4 |
| Normansell R, 2017 | Asthma | Electronic trackers or reminders | No added intervention i.e. “usual care” | Exacerbations requiring OCS (people with at least 1) | \| 307 \| \| --- \| \| 73 \| \| (26-208) \| \|  \| | 0.86  [0.38;1.95] | 17 |
| Chamberlain C, 2017 | Smoking cessation (pregnant women) | All experimental intervention | No added intervention i.e. “usual care” | Abstinence in late pregnancy | \| 6664 \| \| --- \| \| 143,5 \| \| (60-1090) \| \|  \| | 0.59  [0.44;0.8] | 63 |
| Stacey D, 2017 | Decision aids | Decision aid | No added intervention i.e. “usual care” | Accurate risk perceptions | \| 704 \| \| --- \| \| 204 \| \| (170-330) \| \|  \| | 0.07  [0.01;0.51] | 90 |
| Schuit E, 2017 | Smoking cessation | Active NRT | Active comparator | Six-Month Abstinence | \| 552 \| \| --- \| \| 193 \| \| (58-301) \| \|  \| | 0.9  [0.39;2.08] | 48 |
| van Driel ML, 2016 | Lipid-lowering medication adherence | Intensified | No added intervention i.e. “usual care” | Medication adherence at ≤ 6 months | \| 5988 \| \| --- \| \| 198 \| \| (30-4548) \| \|  \| | 0.51  [0.29;0.89] | 82 |
| Linde K, 2016 | Prevention of tension type headache | Acupuncture | Sham comparator | Response | \| 703 \| \| --- \| \| 132 \| \| (30-409) \| \|  \| | 0.63  [0.46;-0.85] | 0 |
| Heal CF, 2016 | Preventing surgical site infection | Topical antibiotic | Placebo/no treatment | Surgical site infection | \| 2310 \| \| --- \| \| 327 \| \| (92-972) \| \|  \| | 0.46  [0.33;0.65] | 0 |
| Heneghan CJ, 2016 | Self-management of oral anticoagulation | Self-management | No added intervention i.e. “usual care” | Major haemorrhage | \| 2233 \| \| --- \| \| 317,5 \| \| (49-737) \| \|  \| | 0.68  [0.42;1.08] | 0 |
| Stead LF, 2016 | Smoking cessation | Combined intervention | Usual care/active control | Cessation at longest follow up | \| 7186 \| \| --- \| \| 205 \| \| (64-1207) \| \|  \| | 0.46  [0.36;0.6] | 51 |
| Posadzki P, 2016 | Management of long term conditions | ATSC | Other/no call/usual care | Smoking abstinence | \| 2145 \| \| --- \| \| 725 \| \| (397-1023) \| \|  \| | 0.75  [0.57;0.98] | 34 |
| Martineau AR, 2016 | Asthma | Vitamin D | Placebo | People with one or more exacerbations requiring systemic corticosteroids | \| 933 \| \| --- \| \| 89 \| \| (22-408) \| \|  \| | 0.76  [0.52;1.13] | 0 |
| Petsky HL, 2016 | Asthma | FeNO strategy | No added intervention i.e. “usual care” | Number of participants who had ≥ 1 exacerbations over study period | \| 911 \| \| --- \| \| 200,5 \| \| (118-392) \| | 0.54  [0.4;0.73] | 0 |
| Kew KM, 2016 | Asthma | Telemonitoring + feedback | No added intervention i.e. “usual care” | Exacerbations requiring hospital admission | \| 795 \| \| --- \| \| 78 \| \| (44-281) \| \|  \| | 0.57  [0.23;1.43] | 26 |
| Venekamp RP, 2015 | Acute otitis media in children | ATB | Placebo | Pain | \| 947 \| \| --- \| \| 232,5 \| \| (165-317) \| \|  \| | 0.92  [0.45;1.87] | 57 |
| Reilly S, 2015 | People with dementia | Case management | No added intervention i.e. “usual care” | Institutionalised | \| 317 \| \| --- \| \| 102 \| \| (81-134) \| \|  \| | 2.49  [0.66;9.49] | 0 |
| \| **Hilton MP, 2014** \| \| --- \| \|  \| \|  \| \|  \| | Benign paroxysmal positional vertigo | Epley | Placebo | Complete resolution of vertigo symptoms | \| 273 \| \| --- \| \| 50 \| \| 33 \| \| (79-273) \| \|  \| | 0.2  [0.07;0.57] | 71 |
| Zwerink M, 2014 | COPD | Self-management | No added intervention i.e. “usual care” | Respiratory-related hospital admissions | \| 189 \| \| --- \| \| 75 \| \| (38-76) \| \|  \| | 0.99  [0.36;2.73] | 0 |
| Purgato M, 2014 | Depression | Paroxetine | Active comparator | Failure to respond at endpoint | \| 1093 \| \| --- \| \| 90 \| \| (40-217) \| \|  \| | 1.03  [0.81;1.31] | 0 |
| Venekamp RP, 2014 | Acute sinusitis | Oral corticosteroids | Placebo/active comparator | Proportion of patients with resolution or improved symptoms | \| 1043 \| \| --- \| \| 203 \| \| (42-417) \| \|  \| | 0.48  [0.3;0.78] | 66 |
| Stead LF, 2013 | Smoking cessation | Physician advice | No added intervention i.e. “usual care” | smoking cessation at longest follow up | \| 11870 \| \| --- \| \| 530 \| \| (191-3128) \| \|  \| | 0.5  [0.36;0.68] | 51 |
| Kenealy T, 2013 | Common cold and acute purulent rhinitis | Antibiotic | Placebo | Persisting symptoms 1 to 7 days | \| 637 \| \| --- \| \| 261 \| \| (88-288) \| \|  \| | 2.16  [0.51;9.09] | 83 |
| Gurol‐Urganci I, 2013 | Attendance at healthcare appointments | SMS reminders | No added intervention i.e. “usual care” | Attendance rate at healthcare appointments | \| 3609 \| \| --- \| \| 664 \| \| (415-1234) \| \|  \| | 0.69  [0.56;0.84] | 33 |
| Timmer A, 2013 | Acute respiratory tract infections | p. sidoides | Placebo | Failure to resolve key symptom by day seven: sputum | \| 746 \| \| --- \| \| 135 \| \| (124-217) \| \|  \| | 0.37  [0.26;0.54] | 15 |
| Dennis CL, 2013 | Post-partum depression | All psychosocial and psychological interventions | No added intervention i.e. “usual care” | Depressive symptomatology at final study assessment | \| 5378 \| \| --- \| \| 353 \| \| (103-1401) \| \|  \| | 0.62  [0.42;0.92] | 79 |
| Magni LR, 2013 | Depression | Fluoxetine | Active comparator | Failure to respond | \| 446 \| \| --- \| \| 84 \| \| (26-157) \| \|  \| | 1.09 [0.62;1.9] | 44 |
| Koning S, 2012 | Impetigo | Topical antibiotic | Placebo | Cure/improvement | \| 293 \| \| --- \| \| 102 \| \| (35-156) \| \|  \| | 0.15  [0.09;0.26] | 0 |
| Gillespie LD, 2012 | Prevention of falls in older people | Multifactorial intervention | No added intervention i.e. “usual care” | Number of fallers | \| 2890 \| \| --- \| \| 272,5 \| \| (92-650) \| \|  \| | 0.94  [0.67;1.32] | 69 |
| Sheikh A, 2012 | Acute bacterial conjunctivitis | Antibiotic | Placebo | Microbiological remission | \| 443 \| \| --- \| \| 143 \| \| (50-250) \| \|  \| | 0.41  [0.21;0.8] | 55 |
| De Sutter AIM, 2012 | Common cold | Antihistamine-decongestant | Placebo/active comparator | Global evaluation | \| 403 \| \| --- \| \| 116 \| \| (83-204) \| \|  \| | 3.41  [1.35;8.6] | 76 |
| Leucht C, 2012 | Major depressive disorder | Amitriptyline | Placebo | Response | \| 251 \| \| --- \| \| 47 \| \| (20-100) \| \|  \| | 0.28  [0.16;0.5] | 0 |
| Kwok CS, 2012 | Cutaneous warts | Topical salicylic acid | Placebo/no treatment | Cure rate all studies all sites | \| 486 \| \| --- \| \| 56,5 \| \| (20-164) \| \|  \| | 0.35  [0.22;0.54] | 4 |
| Edmonds ML, 2012 | Asthma | ICS | Active comparator | Asthma relapse | \| 684 \| \| --- \| \| 129,5 \| \| (22-403) \| \|  \| | 1  [0.66;1.52] | 0 |
| Ruepert L, 2011 | Irritation bowel syndrome | Antidepressant | Placebo | successfully treated patients with IBS | \| 870 \| \| --- \| \| 103 \| \| (68-233) \| \|  \| | 0.2  [0.09;0.47] | 62 |
| Watanabe N, 2011 | Depression | Mirtazapine | Active comparator | Response at the end of the acute phase treatment | \| 1226 \| \| --- \| \| 197 \| \| (40-251) \| \|  \| | 0.91 [0.72;1.14] | 0 |
| Everett T, 2011 | Cervical screening | Invitation | No added intervention i.e. “usual care” | Uptake of screening | \| 3004 \| \| --- \| \| 354 \| \| (196-1794) \| \| 1794 \| | 0.54  [0.44;0.67] | 0 |
| Cahill K, 2010 | Smoking cessation | Any staged intervention | No added intervention i.e. “usual care” | Abstinence at longest follow up | \| 2629 \| \| --- \| \| 518,5 \| \| (93-1499) \| \|  \| | 0.48  [0.26;0.89] | 53 |
| Omori IM, 2010 | Depression | Fluvoxamine | Active comparator | Response (acute phase) | \| 345 \| \| --- \| \| 63,5 \| \| (23-86) \| \|  \| | 0.88  [0.55;1.39] | 0 |
| Kilburn SA, 2010 | Cellulitis and erysipelas | Newer cephalosporin | Active comparator | Symptom free/reduced at the end of treatment | \| 153 \| \| --- \| \| 45 \| \| (34-74) \| \|  \| | 1.22  [0.37;3.99] | 0 |
| Linde K, 2008 | Depression | Hypericum | Placebo | Responder | \| 338 \| \| --- \| \| 97 \| \| (88-153) \| \|  \| | 0.47  [0.29;0.74] | 0 |
| Hunot V, 2007 | Generalised anxiety disorders | Psychological therapies | No added intervention i.e. “usual care” | Clinical response at post-treatment | \| 159 \| \| --- \| \| 49 \| \| (30-80) \| \|  \| | 5.47  [2.36;12.68] | 0 |
| Guaiana G, 2007 | Depression | Amitriptyline | Active comparator | Responders (ITT) | \| 2050 \| \| --- \| \| 48 \| \| (14-365) \| \|  \| | 0.77  [0.6;1] | 31 |
| Gilbody S, 2005 | Depression | Management of depression following feedback | No added intervention i.e. “usual care” | Any intervention for depression | \| 1208 \| \| --- \| \| 221 \| \| (88-454) \| \|  \| | 0.58  [0.25;1.33] | 89 |
| Furukawa TA, 2003 | Depression | Low dosage TCA | Placebo | Depression improved | \| 151 \| \| --- \| \| 37,5 \| \| (22-54) \| \|  \| | 0.66  [0.33;1.3] | 0 |
| van Tulder MW, 2003 | Low back pain | Muscle relaxant (non BZD) | Placebo | Pain | \| 254 \| \| --- \| \| 108 \| \| (30-116) \| \|  \| | 0.51  [0.13;2.05] | 61 |
| Gibson PG, 2002 | Asthma | Self-management | No added intervention i.e. “usual care” | ER Visits | \| 1551 \| \| --- \| \| 211 \| \| (56-593) \| \|  \| | 0.95  [0.71;1.26] | 0 |
| DiGuiseppi C, 2001 | Smoking cessation | Smoke alarm | No added intervention i.e. “usual care” | Final smoke alarm ownership | \| 1783 \| \| --- \| \| 358 \| \| (123-944) \| \|  \| | 0.82  [0.38;1.79] | 68 |
