# Supplementary material for: Intervention effect estimates in randomised controlled trials conducted in primary care versus secondary or tertiary care settings: a meta-epidemiological study
Source: BMC Med Res Methodol. 2022 Dec 22;22:329. doi: 10.1186/s12874-022-01815-2 (PMC9773496; doi:10.1186/s12874-022-01815-2)
Supplement: Supplementary file 3 — Additional file 3. Characteristics of the primary care setting randomised controlled trials included. [file 12874_2022_1815_MOESM3_ESM.docx]

**Additional File 3.** Characteristics of the primary care setting randomised controlled trials included

| **PC-RCTs characteristics** | **Total = 230** |
| --- | --- |
|  | N (%) |
| **Settings** |  |
| Community pharmacy | 6 (2.6) |
| Community health center | 3 (1.3) |
| Family practice | 21 (9.1) |
| General practice | 79 (34.3) |
| Primary care | 25 (10.9) |
| Primary care clinic | 34 (14.8) |
| Primary care practice | 20 (8.7) |
| Via medical record or list | 3 (1.3) |
| Other | 39 (17.0) |
| **Continents** |  |
| Africa | 1 (0.4) |
| North America | 63 (27.4) |
| Asia | 8 (3.5) |
| Europe | 116 (50.4) |
| United-Kingdom | 49 (21.3) |
| France | 2 (0.9) |
| Germany | 9 (3.9) |
| Netherland | 16 (7.0) |
| Other | 40 (17.4) |
| Oceania | 16 (7.0) |
| Not mentionned | 26 (11.3) |
| **Healthcare professionals** |  |
| General practitionner | 16 (7.0) |
| Pharmacist | 4 (1.7) |
| Practice nurse | 1 (0.4) |
| Others | 10 (4.3) |
| Not mentionned | 199 (86.5) |
| **Settings where patients are seen**  **during the study for recruitment,**  **follow-up and primary outcome**  **assessment** | |
| Yes | 15 (6.5) |
| No | 62 (27.0) |
| Unclear | 153 (66.5) |

PC-RCTs: primary care setting randomised controlled trials
